# Inflammatory and Immune Proteins in Umbilical Cord Blood: Association with Hearing Screening Test Failure in Preterm Neonates

**DOI:** 10.1155/2018/4209359

**Published:** 2018-09-19

**Authors:** Ye Ji Shim, Byung Yoon Choi, Kyo Hoon Park, Hyunju Lee, Young Mi Jung, Yu Mi Kim

**Affiliations:** ^1^Department of Otorhinolaryngology–Head and Neck Surgery and Healthcare Research Institute, Seoul National University Hospital, Healthcare System Gangnam Center, Seoul, Republic of Korea; ^2^Department of Otorhinolaryngology–Head and Neck Surgery, Seoul National University College of Medicine, Seoul National University Bundang Hospital, Seongnam, Republic of Korea; ^3^Department of Obstetrics and Gynecology, Seoul National University College of Medicine, Seoul National University Bundang Hospital, Seongnam, Republic of Korea; ^4^Department of Pediatrics, Seoul National University College of Medicine, Seoul National University Bundang Hospital, Seongnam, Republic of Korea

## Abstract

**Objective:**

We aimed to determine whether elevated levels of various inflammatory and immune proteins in umbilical cord blood are associated with an increased risk of newborn hearing screening (NHS) test failure in preterm neonates.

**Methods:**

This retrospective cohort study included 127 premature singleton infants who were born at ≤33.6 weeks. Umbilical cord plasma at birth was assayed for interleukin (IL)-6, complement C3a and C5a, matrix metalloproteinase (MMP)-9, macrophage colony-stimulating factor (M-CSF), and endostatin levels using ELISA kits. Neonatal blood C-reactive protein (CRP) levels were measured within 2 hours of birth. The primary outcome measure was a uni- or bilateral refer result on an NHS test. Univariate and multivariate analyses were applied.

**Results:**

Fifteen (11.8%) infants failed the NHS test. In the univariate analyses, high IL-6 and low C3a levels in umbilical cord plasma, funisitis, and an elevated CRP level (>5 mg/L) in the immediate postnatal period were significantly associated with NHS test failure. However, the levels of umbilical cord plasma MMP-9, C5a, M-CSF, and endostatin were not significantly different between infants who passed and those who failed the NHS test. Multiple logistic regression analyses indicated that elevated umbilical cord plasma C3a levels were independently associated with a reduced risk of NHS test failure, whereas elevated levels of umbilical cord plasma IL-6 and high CRP levels in the immediate postnatal period were significantly associated with NHS test failure.

**Conclusions:**

Our data demonstrated that in preterm neonates, a systemic fetal inflammatory response reflected by umbilical cord plasma IL-6 and immediate postnatal CRP levels may contribute to the risk for NHS test failure, whereas the changes in complement activation fragments initiated *in utero* may have protective effect of hearing screen failure.

## 1. Introduction

Sensorineural hearing loss (SNHL) is one of the most common long-term disabilities worldwide in preterm infants, with an incidence of 0.7–17.5% for very preterm newborns (<32 weeks) [1–3]. Given the high prevalence and clinical relevance of SNHL in preterm neonates, the early detection and proper treatment of SNHL are important for normal speech development. Therefore, the identification of biomarkers that can ensure the early identification of preterm neonates at the highest risk of SNHL, thus enabling early therapeutic intervention or auditory rehabilitation such as hearing aid or cochlear implantation, is urgently needed.

Although many perinatal and postnatal factors associated with SNHL have been reported in the literature [1–4], little is known regarding its prenatal risk factors. Importantly, recent studies by Leung et al. and our group have demonstrated that the presence of intra-amniotic infection (with reported incidences of 13.6% for preterm labor and 38% for preterm premature rupture of membranes) [5, 6], funisitis, and fetal inflammatory response syndrome (FIRS, defined as an elevated fetal plasma interleukin-6 level (>11 pg/mL) and/or the presence of funisitis/chorionic vasculitis) [7, 8] were significantly associated with an increased risk of hearing screening failure in very preterm neonates [9, 10], suggesting that infection/inflammation *in utero*, including fetal infection/inflammation, may have a potentially deleterious effect on fetal auditory development. In this regard, an analysis of biomarkers in umbilical cord blood (UCB) may be useful for estimating the risk of SNHL because UCB can directly reflect fetal status, including the effects of the *in utero* milieu on the fetus, such as infection/inflammation, stress, and hypoxia. In fact, several studies have reported a significant association between elevated cytokine levels in the UCB and neurologic disability in very preterm infants [11, 12]. However, only one study to date has examined the relationship between UCB cytokines and hearing screen failure [9]. Moreover, this study was limited because sampling was not necessarily performed immediately after birth from UCB but rather in a broader time window of the 12-hour period after birth from umbilical cord or venous blood, resulting in inadvertent contamination of the results by the effects of postnatal factors [9]. The purpose of this study was to determine whether elevated levels of various inflammatory and immune proteins examined exclusively in the UCB are associated with an increased risk of newborn hearing screening (NHS) test failure in preterm neonates.

## 2. Materials and Methods

### 2.1. Study Design

This single-center retrospective cohort study included infants admitted to the neonatal intensive care unit at Seoul National University Bundang Hospital (Seongnam, Korea) between June 2004 and January 2015. The inclusion criteria were (1) singleton birth at 23^+0^ to 33^+6^ weeks gestation, (2) survival at least 90 days after birth, (3) underwent hearing screening test, and (4) an aliquot of UCB available for analysis. We excluded twins or higher-order infants, those for whom a histologic examination of the placenta was not performed, outborn infants, and those with major structural or chromosomal abnormalities. Gestational age was calculated based on the last menstrual period and ultrasound information obtained in the first or second trimester. The study was approved by the local ethics committee of Seoul National University Bundang Hospital (IRB no. B-1006/103-102). Written informed consent was obtained from the parents of all infants (participants) whose samples and data were used for the study.

### 2.2. Hearing Screening

Electronic medical records on uni- or bilateral hearing screen failure of the included preterm singleton infants were reviewed by one otolaryngologist (Y. J. S.) who was blinded to the results of umbilical cord plasma analysis and the details of mothers and their infants. The conventional methods for hearing screening and the follow-up in our hospital were previously described in detail elsewhere [10, 13]. In brief, the automated auditory brainstem response (AABR) test was the most commonly performed (*n* = 107) NHS test, while the otoacoustic emission (OAE) test was performed in 20 cases in which the AABR was not available. If the infant failed either the AABR or OAE test, the same test was repeated. Infants who failed two consecutive screenings of the AABR or OAE in one or both ears were classified as hearing screen failure. The results were recorded as either “refer” (further confirmation tests, such as an auditory brainstem response threshold test, needed) or “pass” (normal). Therefore, the primary outcome measure was a uni- or bilateral refer result on an NHS test.

### 2.3. Clinical Data and Definitions of Risk Factors for Hearing Screen Failure

The following maternal factors were extracted from the database: maternal age, parity, gestational age at admission, causes of preterm birth, delivery mode, antenatal use of medications (tocolytics, steroids, and antibiotics), and clinical diagnosis of chorioamnionitis. Perinatal/neonatal characteristics retrieved from the database were as follows: gestational age at birth, sex, birth weight, 1 and 5 min Apgar scores, pathologic diagnoses of the placenta, umbilical artery pH, neonatal blood C-reactive protein (CRP) levels and white blood cell (WBC) counts obtained within 2 hours of birth, use of surfactant, use of mechanical ventilation, proven neonatal sepsis, respiratory distress syndrome (RDS), bronchopulmonary dysplasia (BPD), intraventricular hemorrhage (IVH), periventricular leukomalacia (PVL), and necrotizing enterocolitis (NEC).

Clinical chorioamnionitis was diagnosed in accordance with the criteria of Gibbs et al. [14]. Proven sepsis, RDS, BPD, IVH, PVL, and NEC were diagnosed according to the definitions previously described in detail [10, 15]. Acute histologic chorioamnionitis was defined as the presence of an acute inflammatory change in any tissue sample (amnion, chorion-decidua, umbilical cord, or chorionic plate) using the criteria published previously [16]. Funisitis was diagnosed by the presence of neutrophil infiltration into the umbilical vessel walls or Wharton's jelly. Fetal inflammatory response syndrome (FIRS) is defined as the presence of funisitis or elevated levels of umbilical cord plasma IL-6 (>11 pg/mL) [7, 17]. Neonatal blood CRP levels up to 2 hours postdelivery were analyzed as categorical variables because several CRP measurements were performed qualitatively and grouped by value (>5 vs ≤5 mg/L). Thus, a CRP level > 5 mg/L was considered elevated; that is, it exceeded the 95^th^ percentile for CRP at birth based on the data for healthy term and near-term infants [18].

### 2.4. Analysis of Inflammatory-Related Proteins in the Umbilical Cord Plasma

UCB samples were obtained from the umbilical vein at birth and collected into ethylenediaminetetraacetic acid tubes. The samples were centrifuged at 1500 × *g* at 4°C for 10 minutes, and the supernatant was aliquoted and stored at −70°C until being assayed. The stored plasma samples were assayed for multiple inflammatory and immune proteins (interleukin (IL)-6, complements C3a and C5a, matrix metalloproteinase (MMP)-9, macrophage colony-stimulating factor (M-CSF), and endostatin). Enzyme-linked immunosorbent assay kits were used to measure IL-6 (R&D Systems, Minneapolis, MN, USA), MMP-9, M-CSF, and endostatin (DuoSet ELISA; R&D Systems) and complement C3a and C5a (BD Biosciences, San Diego, CA, USA) levels in the umbilical cord plasma samples according to the manufacturers' instructions. The ranges of the IL-6, C3a, C5a, MMP-9, M-CSF, and endostatin standard curves were 0.2–10 pg/mL, 0.078–2.5 ng/mL, 0.08–2.5 ng/mL, 31.2–2000 pg/mL, 62.50–4000 pg/mL, and 62.50–4000 pg/mL, respectively. Prior to the measurement of these six proteins, the umbilical cord plasma samples were diluted using the ratio 1 : 5 for IL-6, 1 : 10 for C5a and M-CSF, 1 : 100 for MMP-9 and endostatin, and 1 : 5000 for C3a. In the samples with protein concentrations lower than the lowest point on the standard curve, the lowest detected values were used for the analysis. The intra- and interassay coefficients of variation were 4.0% and 12.2% for IL-6, 5.5% and 18.5% for C3a, 6.9% and 12.6% for C5a, 3.1% and 7.0% for MMP-9, 2.7% and 9.7% for M-CSF, and 1.5% and 10.9% for endostatin, respectively.

### 2.5. Statistical Analysis

Statistical analyses were performed using SPSS version 22.0 for Windows (IBM SPSS Statistics, Chicago, IL, USA). The Shapiro-Wilk test was used to assess whether the data were normally distributed. For the bivariate analyses, Student's *t*-test or the Mann-Whitney U test was used for continuous data, while the *χ*^2^ test or Fisher's exact test was used to examine categorical data. A multivariate logistic regression model was further performed to examine the relationship of the level of each protein in umbilical cord plasma to the failure in the NHS test after adjusting for baseline variables. Variables with a *P* value < 0.05 on the bivariate analyses were included in the logistic regression analysis. Receiver operating characteristic (ROC) curves analyses were performed of each protein in the umbilical cord plasma for predicting NHS failure and identifying the best cutoff values for each variable. The Spearman rank correlation test was used to measure the relationship between nonnormally distributed continuous variables. All statistical analyses were performed using a two-sided test with a significance level of 0.05.

## 3. Results

During the study period, a total of 127 women with either preterm labor (*n* = 54) or preterm premature rupture of membranes (*n* = 73) and their neonates who met the inclusion criteria were ultimately included in the analysis. The mean gestational age at birth of the cohort was 30.7 weeks (SD, 1.9 weeks; range, 24.5–33.5 weeks), and the mean birth weight was 1627 g (SD, 431 g; range, 700–2620 g). One hundred twelve neonates (88.2%) passed the NHS test bilaterally, whereas 15 (11.8%) failed the NHS test. Among those 15 neonates, 10 (7.8%) had unilateral failure, while the other five (3.9%) had bilateral failure. Breaking down the failure cases in terms of the screening tests, nine ears of seven neonates (4.20% (9/214) and 6.54% (7/107)) had a “refer” result on the automated ABR, while 11 ears of eight neonates (27.5% (11/40) and 40% (8/20)) had a “refer” result on the automated OAE.

### 3.1. Univariate Relationship of Clinical and Laboratory Factors with Hearing Screen Failure

The maternal and obstetric characteristics of the study population according to NHS test results are shown in Table 1. Mothers delivering neonates with a refer result on the NHS test had a significantly higher rate of funisitis (*P* = 0.043) and tended to have a higher tendency of clinical chorioamnionitis (*P* = 0.078) than mothers who delivered neonates who passed the NHS tests. However, there were no significant intergroup differences in maternal demographics, antenatal medications, or the rate of histologic chorioamnionitis.

The proportions of umbilical cord plasma samples with detectable protein levels were 98.4% for M-CSF and 100% for C3a, C5a, IL-6, MMP-9, and endostatin. Of these six proteins measured in the umbilical cord plasma, MMP-9 levels were significantly positively correlated with those of all proteins but M-CSF (*r* = 0.233–0.342, *P* < 0.01), whereas endostatin levels were significantly positively correlated with all proteins but C3a and C5a (*r* = 0.198–0.236, *P* < 0.05). M-CSF levels in the umbilical cord plasma were significantly correlated with endostatin levels only (*r* = 0.227, *P* = 0.01). Regarding the correlation between C3a, C5a, and IL-6 levels in the umbilical cord plasma, positive significant correlations were found only between C3a and C5a (*r* = 0.401, *P* < 0.001) and between C5a and IL-6 (*r* = 0.264, *P* = 0.003).

Table 2 shows the umbilical cord plasma levels of the inflammatory and immune proteins by NHS test results. Neonates who failed the NHS test had a significantly higher median umbilical cord plasma IL-6 level and lower median umbilical cord plasma C3a level than neonates who passed the NHS test. However, there were no significant intergroup differences in umbilical cord plasma C5a, MMP-9, M-CSF, and endostatin levels, FIRS rates, or elevated umbilical cord plasma IL-6 (>11 pg/mL) levels.

Table 3 shows the neonatal characteristics by NHS test results. The rate of an elevated CRP level (>3 mg/L) in the immediate postnatal period was significantly higher in the neonates who failed the NHS test than those who passed the NHS test. However, no significant differences were found between neonates who passed and those who failed the NHS test in terms of neonatal characteristics and morbidities, including gestational age at birth, umbilical artery pH, major treatments (i.e., continuous positive airway pressure, mechanical ventilation, and surfactant use), and major neonatal morbidities (i.e., proven sepsis, RDS, BPD, IVH, PVL, or NEC).

### 3.2. Multivariate Analysis

Multiple logistic regression analyses were performed to further examine the relationship between the various proteins in the umbilical cord plasma and NHS test failure after adjusting for the effects of baseline variables. The following variables were assessed in the multivariate logistic regression analysis as significant predictors in the univariate analyses **(***P* < 0.05): umbilical cord plasma IL-6 and C3a levels, funisitis, and an elevated CRP level (>3 mg/L) in the immediate postnatal period. Prior to performing logistic regression analysis for testing the model, tests for multicollinearity among the independent variables were performed using bivariate analyses (e.g., *χ*^2^ test, Spearman's rank correlation test, and the Mann-Whitney U test). Significant correlations were found among funisitis, umbilical cord plasma IL-6 levels, and an elevated CRP level in the immediate postnatal period in bivariate analyses (*P* = 0.001 to <0.001), whereas umbilical cord plasma C3a level was correlated with none. Therefore, funisitis, umbilical cord plasma IL-6 levels, and elevated CRP levels in the immediate postnatal period were analyzed in separate models (Table 4). After adjustments for umbilical cord plasma C3a levels, elevated umbilical cord plasma IL-6 level, funisitis, and elevated blood CRP levels in the immediate postnatal period were significantly associated with NHS test failure. When these four variables were simultaneously entered into logistic regression analysis, elevated umbilical cord plasma C3a levels were independently associated with a reduced incidence of NHS test failure (Table 4).

### 3.3. ROC Curve Analysis

**Figure 1:**
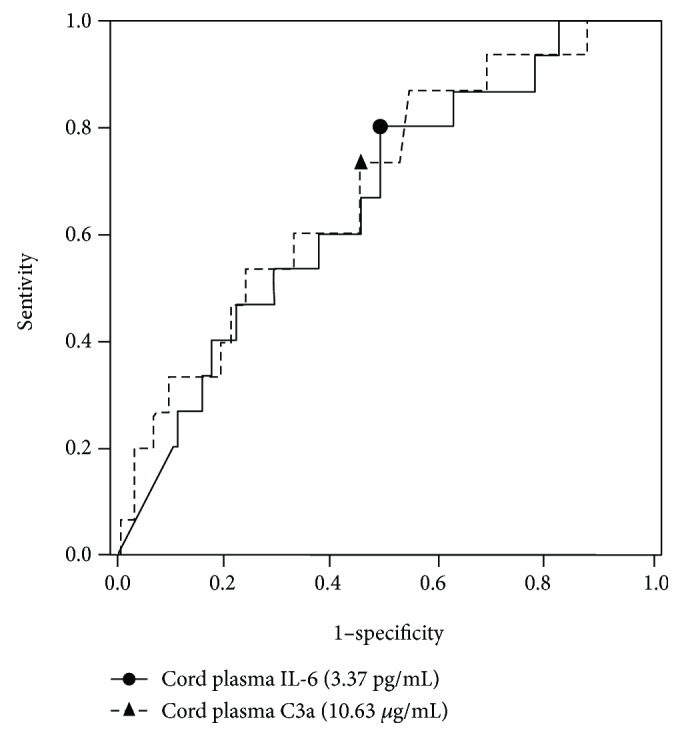
Receiver operating characteristic curves for umbilical cord plasma interleukin-6 (IL-6) “line” and C3a “broken line” for predicting newborn hearing screening test failure (cord plasma IL-6: area under the curve, 0.663; standard error, 0.070; cord plasma C3a: area under the curve, 0.690; standard error, 0.071; no differences (*P* = 0.778) between cord plasma IL-6 and C3a).

Figure 1 displays the ROC curves for the umbilical cord plasma C3a and IL-6 levels in predicting NHS test failure. The area under the curve (AUC) for umbilical cord plasma IL-6 and C3a levels for predicting NHS test failure was 0.663 (95% confidence interval (CI), 0.526–0.801, *P* = 0.040) and 0.690 (95% CI, 0.552–0.829, *P* = 0.017), respectively. The best cutoff values (sensitivity and specificity) for predicting failure in the NHS test were 3.37 pg/mL for umbilical cord plasma IL-6 (80.0% sensitivity, 51.8% specificity) and 10.63 *μ*g/mL for umbilical cord plasma C3a (73.3% sensitivity, 55.4% specificity) (Figure 1).

## 4. Discussion

### 4.1. Principal Findings of This Study

Our data demonstrate that in preterm neonates, a systemic fetal inflammatory response reflected by umbilical cord plasma IL-6 and immediate postnatal CRP levels may contribute to the risk for NHS test failure, whereas the changes in complement activation fragments initiated *in utero* may have protective effect of hearing screen failure. These findings support the hypothesis that a systemic fetal inflammatory response and changes in complement activation fragments initiated *in utero* might be involved in the pathophysiological mechanism of hearing loss in preterm infants and suggest that the optimal timing for therapeutic strategies (e.g., antimicrobial therapy for the prevention and treatment of fetal infection) intended to prevent hearing loss in preterm infants may be prior to delivery.

### 4.2. Meaning of This Study

In the literature, elevated IL-6 UCB levels at birth were reportedly associated with an increased risk of neonatal morbidity and mortality, including neonatal sepsis, systemic inflammatory response syndrome, PVL, and NEC [19–21]. However, little is known about the associations among elevated levels of UCB IL-6, abnormal NHS results, and subsequently confirmed SNHL in preterm infants. In the context of abnormal NHS results, Leung et al. showed an elevated IL-6 level in neonatal blood obtained within 12 hours of birth but not necessarily at birth was a risk factor for hearing screen failure [9]. In accordance with the previous report, our results also demonstrated that elevated IL-6 levels in UCB, reflecting an *in utero* initiation of the fetal response to perinatal events, were significantly associated with NHS test failure. Given that IL-6 is a well-known important mediator of host response to infection [22], which in turn is significantly involved in the pathogenesis of sensorineural hearing impairment [10, 23], these observations are not unexpected and support the hypothesis that a systemic fetal inflammatory response may be detrimental to auditory function in preterm infants.

The complement system plays a central role in innate immunity that provides an effective first line of defense against infection by triggering inflammatory responses [24], but its role in inner ear damage remains unclear. Unexpectedly, the current study showed that elevated levels of umbilical cord plasma C3a were independently associated with a reduced risk of NHS test failure. This finding is similar to the previously reported clinical evidence and animal data on the relationship between neonatal hypoxic–ischemic brain injury and complement peptide C3a, which have shown that C3a protects the brain tissue against neonatal hypoxia–ischemia injury, especially in the immature brain [25, 26]. Indeed, C3a has been shown to have an anti-inflammatory effect [27] and a neuroprotective effect *in vitro* via acting on many types of glia in the central nervous system (e.g., astrocytes, microglia, and oligodendrocytes) [28, 29] corresponding to support cells in the cochlea underlying a subset of pathophysiology of sensorineural hearing loss. Moreover, from a therapeutic perspective, recent studies by Morán et al. and Järlestedt et al. demonstrated that the application of C3a has profound effects on the amelioration of neonatal hypoxic–ischemic brain injury in a mouse model [25, 30]. Therefore, it is likely that C3a could also serve as a potential new therapeutic substance targeting treatment for SNHL. Further studies are needed to determine whether abnormal NHS results and hearing impairment could be reduced by early C3a treatment in an animal model and subsequent clinical trials in preterm neonates at high risk of developing SNHL.

A body of research has suggested that prenatal infection and resultant fetal inflammatory response contribute to the pathogenesis of severe neonatal neurologic illness, such with white matter injury (WMI) [31, 32], which in turn was associated with an increased risk of neurosensory impairment (hearing or vision) [33, 34], and elevated blood CRP levels at birth were associated with WMI in preterm infants [35]. However, to date, no study has evaluated the direct relationship between immediate postnatal blood CRP levels (at or immediately after birth) and hearing status in preterm infants. However, of note, in a study that analyzed the CRP values reflecting rather postnatal inflammatory status due to infection (using the maximum CRP values obtained during the entire course before the auditory screening), Yoshikawa et al. found a significant association between high neonatal blood CRP levels and hearing screen failure [36]. Similar to the findings in this study, we further found that elevated CRP levels in the immediate postnatal period, which may be more reflective of perinatal rather than postnatal events, were also significantly associated with NHS test failure. In fact, these observations are natural because CRP is predominantly secreted by the liver in response to an elevated IL-6 level [37], which was already reported to be a major independent risk factor for fetal and neonatal disorders associated with neonatal hearing impairment, such as FIRS, funisitis, early onset neonatal sepsis, and WMI in preterm infants [9, 10, 21, 33, 38]. It is most likely that the presence of a ripple effect of bacterial and viral infections such as inflammatory reaction in the cochlea as reflected by an increase in CRP levels could cause NHS test failure.

Our failure to obtain a statistical association between NHS test failure and MMP-9, C5a, M-CSF, and endostatin levels in the umbilical cord plasma in the present study merits attention. A famous tissue remodeling gene, the *MMP-9* gene, was previously reported to be significantly upregulated after exogenous trauma to the cochlea such as cochlear implantation and was even qualitatively associated with a change in hearing thresholds after cochlear implantation in guinea pigs [39]. It could be that the expression of the tissue remodeling gene *MMP-9* is more significantly affected by exogenous trauma than endogenous inflammation. Endostatin has been reported to show beneficial effects on the inflammatory disease and even sepsis as previously shown in a septic mouse model and a rheumatoid arthritis model in which endostatin reduced multiple organ dysfunction syndrome due to sepsis and angiogenesis, respectively [40, 41]. However, we could not observe any protective effect of endostatin from hearing loss status in preterm neonates in the present study. The cochlea might have a different milieu for endostatin to play such a role. Alternatively, the main mechanisms of hearing loss from preterm neonates might walk in a pathway less affected by endostatin. The effect of M-CSF on the hearing status in preterm neonates can be complicated. It can serve as a double edge in terms of effect on the neuron, although it has been expected to exert a mostly protective effect [42]. Indeed, a protective effect on the auditory nerve was reported with the application of M-CSF [43]. The association, if any, between hearing loss status in preterm newborns and M-CSF or endostatin awaits the accumulation of further evidence and data.

### 4.3. Strengths and Limitations of the Study

The current study has several limitations. First, this retrospective study was conducted at a single center with a limited number of subjects, limiting our ability to extrapolate our results to the general population. Second, the role of various immune-related proteins in the UCB in the development of SNHL could not be precisely evaluated because of the low prevalence of SNHL. The SNHL incidence in this study (1.5%, 2/127) was in accordance with those of other studies [1, 44]. Third, a full characterization was not performed on pro- and anti-inflammatory cytokines and MMP in the umbilical cord plasma in this study, which is not likely to reflect the entire picture of immune activation related to hearing loss status. The strength of our study is that it is the first to our knowledge to investigate the relationship between the levels of various inflammatory and immune proteins in the UCB and hearing screen failure that places a neonate at risk for SNHL. Finally, six proteins in the umbilical cord plasma were selected in the present study because their expressions are increased during preterm birth–associated inflammatory/immunological responses in the maternal blood, amniotic fluid, or UCB [12, 19, 45–47].

## 5. Conclusions

In conclusion, in preterm newborns, elevated levels of umbilical cord plasma C3a were independently associated with a reduced risk of NHS test failure, whereas elevated levels of umbilical cord plasma IL-6 and elevated CRP levels in the immediate postnatal period were significantly associated with NHS test failure. However, these measures are not sensitive or specific markers for hearing screen failure (Figure 1). Elevated umbilical cord plasma MMP-9, C5a, M-CSF, and endostatin levels were not associated with hearing screen failure. Further large longitudinal studies are needed to assess which time point provides the best predictive value of subsequent hearing impairment using the fetal and neonatal blood samples collected serially at predefined time points.

## Figures and Tables

**Table 1:** Maternal and obstetric characteristics of the study population according to newborn hearing screening test results.

	Abnormal finding on newborn hearing screening test		*P*
	Absent (*n* = 112)	Present (*n* = 15)	
Maternal age (years)	31.6 ± 3.6	31.8 ± 3.8	0.970
Nulliparity	49 (43.8%)	10 (66.7%)	0.107
Membrane status			0.270
Intact membranes	49 (43.8%)	4 (26.7%)	
Preterm PROM	63 (56.3%)	11 (73.3%)	
Cesarean delivery	44 (39.3%)	4 (26.7%)	0.407
Antenatal corticosteroids	107 (95.5%)	13 (86.7%)	0.193
Antenatal antibiotics	87 (77.7%)	13 (86.7%)	0.737
Antenatal tocolytics	91 (81.3%)	12 (80.0%)	1.000
Gestational age at admission (weeks)	29.3 ± 3.3	28.4 ± 3.6	0.356
Histologic chorioamnionitis	74 (66.1%)	10 (66.7%)	1.000
Funisitis	22 (19.6%)	7 (46.7%)	0.043
Clinical chorioamnionitis	5 (4.5%)	3 (20.0%)	0.078

Values are given as mean ± standard deviation or *n* (%). PROM: premature rupture of membranes.

**Table 2:** Umbilical cord plasma levels of inflammatory and immune proteins according to newborn hearing screening test results.

	Abnormal finding in newborn hearing screening test		*P*
	Absent (*n* = 112)	Present (*n* = 15)	
Umbilical cord plasma IL-6 (pg/mL)	11.0 ± 15.1	19.0 ± 18.7	0.040
Umbilical cord plasma C3a (*μ*g/mL)	11.8 ± 5.9	8.0 ± 5.0	0.017
Umbilical cord plasma C5a (ng/mL)	30.3 ± 22.8	23.6 ± 10.8	0.390
Umbilical cord plasma MMP-9 (ng/mL)	108.0 ± 714.0	83.9 ± 71.2	0.124
Umbilical cord plasma M-CSF (pg/mL)	715.4 ± 390.8	749.7 ± 509.5	0.946
Umbilical cord plasma endostatin (ng/mL)	82.9 ± 16.3	85.6 ± 17.4	0.497
Umbilical cord plasma IL-6 > 11 pg/mL	33 (29.5%)	8 (53.3%)	0.063
Fetal inflammatory response syndrome^a^	43 (38.4.0%)	9 (60.0%)	0.110

Values are given as mean ± standard deviation or *n* (%). IL: interleukin; MMP: matrix metalloproteinase; M-CSF: macrophage colony-stimulating factor. ^a^Fetal inflammatory response syndrome is defined as the presence of funisitis or elevated levels of umbilical cord plasma IL-6 (>11 pg/mL).

**Table 3:** Neonatal characteristics and morbidities according to newborn hearing screening test results.

	Abnormal finding on newborn hearing screening test		*P*
	Absent (*n* = 112)	Present (*n* = 15)	
Gestational age at birth (weeks)	30.8 ± 2.1	30.0 ± 2.9	0.448
Birth weight (kg)	1.6 ± 0.4	1.5 ± 0.5	0.372
Male gender	62 (55.4%)	10 (66.7%)	0.406
Apgar score < 7			
1 min	67 (59.8%)	11 (73.3%)	0.403
5 min	22 (19.6%)	5 (33.3%)	0.310
Umbilical artery pH	7.3 ± 0.06	7.3 ± 0.07	0.671
CRP level > 5 mg/L in immediate postnatal period	8 (7.1%)	4 (26.6%)	0.015
WBC count in immediate postnatal period (10^3^ cells/mm^3^)	12.9 ± 6.8	14.1 ± 14.7	0.625
Continuous positive airway pressure	68 (60.7%)	12 (80%)	0.168
Mechanical ventilation	47 (42.0%)	9 (60.0%)	0.186
Use of surfactant	27 (24.1%)	7 (46.7%)	0.116
Proven sepsis	4 (3.6%)	1 (6.7%)	0.472
Respiratory distress syndrome	39 (34.8%)	7 (46.7%)	0.370
Bronchopulmonary dysplasia	24 (21.4%)	5 (33.3%)	0.331
Intraventricular hemorrhage, grade 2 or more	5 (4.5%)	1 (6.7%)	0.537
Periventricular leukomalacia	9 (8.0%)	1 (6.7%)	1.000
Necrotizing enterocolitis	6 (5.4%)	0 (0.0%)	1.000

Values are given as mean ± standard deviation or *n* (%). CRP: C-reactive protein; WBC: white blood cell.

**Table 4:** Risk factors associated with newborn hearing screening test failure according to logistic regression analyses.

Risk factors	Risk of failure in the newborn hearing screening test			
	Adjusted for umbilical cord plasma C3a		Adjusted for all variables in the model	
	OR (95% CI)	*P*	OR (95% CI)	*P*
Umbilical cord plasma IL-6 (pg/mL)	1.031 (1.000–1.063)	0.050	1.004 (0.960–1.050)	0.859
Funisitis	3.827 (1.197–12.233)	0.024	2.683 (0.694–10.375)	0.153
Elevated blood CRP levels (>5 mg/L) in immediate postnatal period	6.515 (1.814–23.398)	0.019	3.503 (0.524–23.404)	0.196
Umbilical cord plasma C3a (*μ*g/mL)			0.875 (0.780–0.982)	0.023

OR: odds ratio; CI: confidence interval; IL: interleukin; CRP: C-reactive protein.

## Data Availability

The data used to support the findings of this study are available from the corresponding author upon request.
